# Optical Fiber Sensor for Curvature and Temperature Measurement Based on Anti-Resonant Effect Cascaded with Multimode Interference

**DOI:** 10.3390/s22218457

**Published:** 2022-11-03

**Authors:** Yinqiu Gui, Qian Shu, Ping Lu, Jiajun Peng, Jiangshan Zhang, Deming Liu

**Affiliations:** 1 Wuhan National Laboratory for Optoelectronics (WNLO) and National Engineering Laboratory for Next Generation Internet Access System, School of Optical and Electronic Information, Huazhong University of Science and Technology, Wuhan 430074, China; 2 Shenzhen Huazhong University of Science and Technology Research Institute, Shenzhen 518057, China; 3 Wuhan OV Optical Networking Technology Co., Ltd., Wuhan 430074, China; 4 Department of Electronics and Information Engineering, Huazhong University of Science and Technology, Wuhan 430074, China

**Keywords:** fiber optics sensors, integrated optics devices, optical sensing and sensors

## Abstract

In this paper, a novel inline optical fiber sensor for curvature and temperature measurement simultaneously has been proposed and demonstrated, which can measure two parameters with very little crosstalk. Two combinational mechanisms of anti-resonant reflecting optical waveguide and inline Mach–Zehnder interference structure are integrated into a 3 mm-long single hole twin suspended core fiber (SHTSCF). The 85 μm hole core gives periodic several dominant resonant wavelengths in the optical transmission spectrum, acting as the anti-resonant reflecting optical waveguide (ARROW). The modes in two suspended cores and the cladding form the comb pattern. Reliable sensor sensitivity can be obtained by effective experiments and demodulation. Through intensity demodulation of the selected dip of Gaussian fitting, the curvature sensitivity can be up to −7.23 dB/m^−1^. Through tracking the MZI dip for wavelength demodulation, the temperature sensitivity can be up to 28.8 pm/°C. The sensor is simple in structure, compact, and has good response, which can have a bright application in a complex environment.

## 1. Introduction

Fiber optics sensors have been widely used in civil engineering, chemistry and chemical engineering, clinical medicine, power system monitoring and various industries due to their small structure, light weight, and anti-electromagnetic interference characteristics. Especially, optical fiber curvature sensors have outstanding performance in quality monitoring, disaster warning, distributed sensing applications, etc. To date, a variety of optical fiber structures have been proposed and reported for curvature measurement, including fiber gratings [1,2,3], interferometers [4,5,6] and special fiber devices [7,8,9]. However, in practical applications, the optical fiber curvature sensing will be interfered with by physical parameters of the external environment such as temperature. The common method to eliminate temperature crosstalk is to use the sensitivity of different interference peaks and the crossing matrix method [10,11], but it also has cross interference and is difficult to distinguish the dual parameters. Another method is to use a cascade structure [12,13] which has been reported many times, for example, some inline interference structures cascaded with fiber Bragg grating (FBG) are adopted. In some sensing structures, in order to form a variety of interference mechanisms, the sensing part is cascaded with more than two segments, which requires more strict length control and higher production requirements.

Multi-core optical fiber [14], dual-core fiber [15], hollow-core fiber [16,17,18], photonic crystal fiber [19,20] and many other special optical fibers of various structures have also been widely used for curvature and temperature sensing. Among them, hollow-core fiber received widespread attention with its special optical transmission mechanism and has been reported for displacement [21], liquid level [22], and magnetic field sensing [23], while most detection methods only detect one parameter and are easily affected by other parameters. Therefore, it is important to study the measurement dual parameters with little crosstalk. A sensing method that combines a special anti-resonance mechanism, an inline interference structure and a variety of demodulation can effectively avoid the influence of temperature.

In this paper, we proposed a novel type of optical fiber sensor based on the inline Mach–Zehnder interference (MZI) and anti-resonant reflecting optical waveguide (ARROW). The special optical fiber used is a single hole twin suspended core fiber (SHTSCF, YOEC, 125 μm), the big hollow core and suspended core structure of which make the spectrum mainly include two parts, the periodic resonance loss corresponding to the hollow core anti-resonance effect and the comb spectrum corresponding to MZI. Intensity demodulation and wavelength demodulation are used to realize the sensing of curvature and temperature, respectively, which means the wavelength drift change of MZI is mainly affected by temperature, and the intensity change of resonant wavelength is mainly affected by curvature. Hence, two demodulation methods can be used to measure the above two parameters without cross-interference. The length of the sensor is only 3 mm, which can be applied to various complex scenarios due to its robust structure, small size and easy fabrication.

## 2. Sensor Principle and Fabrication

**Figure 1:**
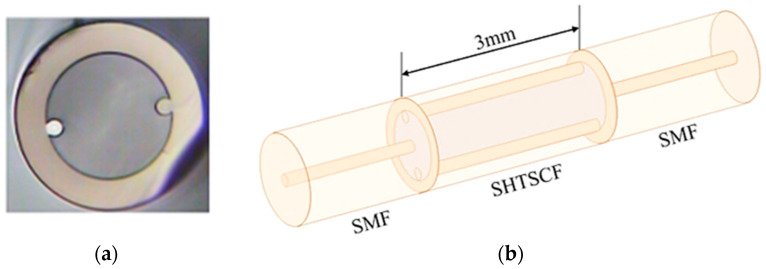
Schematic diagram of the SHTSCF (**a**) Cross-sectional microscopic magnified image of SHTSCF; (**b**) Schematic of sensor structure.

The special optical fiber used in the sensor proposed in this experiment is a single hole twin suspended core fiber (SHTSCF), the cross-sectional view of which is shown in Figure 1a. Different from ordinary single mode fiber (SMF), the center of this special fiber is changed into a large air channel with a diameter of 85 μm. Two identical cores are symmetrically suspended on the wall of the air hole, each with a diameter of 9.1 μm. Surrounded by a solid cladding, the overall diameter of the fiber is 125 μm. The Schematic of the sensor structure is shown in Figure 1b, a section of SHTSCF with a length of 3 mm is fusion spliced between two SMFs; the two fusion points are designed to be partially collapsed. Using the standard SMF-HCF mode on the fusion splicer (Fujikura, FSM-60S, Tokyo, Japan), change the discharge power to standard +10 bit, and the discharge time is 1500 ms. Adjust to manual mode, slightly offset the discharge electrode to the side of SMFs, and there is no gap between the SMF and the SHTSCF.

**Figure 2:**
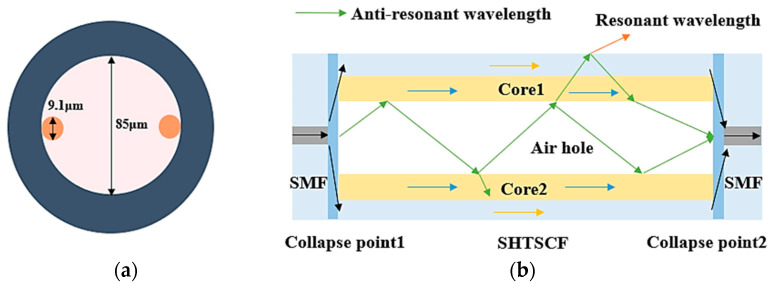
(**a**) The aperture size of the SHTSCF; (**b**) Guiding mechanism of the SHTSCF.

The optical transmission mechanism of SHTSCF can be explained by ARROW, and the anti-resonant effect and beam propagation path are exhibited in Figure 2b. When the beam is transmitted in the air hole, as the refractive index of the core is less than that of the cladding; part of the transmitted light beams will leak into the cladding and then leak out to the external environment at the resonant wavelength. Part of the light beams far away from the resonant wavelength will be confined in the air core to continue forward, that is anti-resonance light. Meanwhile, the two collapse points can play a role in splitting and coupling the transmitted light, where the inline MZI comes into being. According to the optical fiber mode coupling theory, there is also a fiber core mode inside the two suspended fiber cores. Cladding and core modes are excited to form multimode interference. In summary, there are two effects in the light transmission mechanism of the sensor, namely, the inter-mode interference effect formed by multiple modes and the anti-resonant effect caused by the low-refractive index air core.

**Figure 3:**
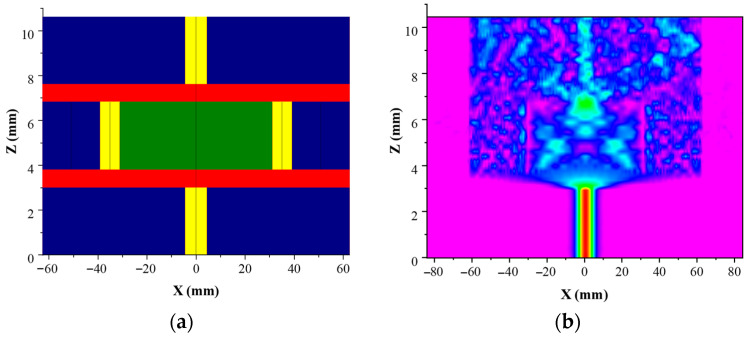
(**a**) Schematic diagram of the establishment of simulation model; (**b**) Simulation of beam propagation at the wavelength of 1550 nm.

In order to verify the mechanism of light transmission in SHTSCF, the BMP module of the optical simulation software RSOFT was used to draw the plane structure diagram of the SMF-SHTSCF-SMF three-layer sensing structure in this paper, as shown in Figure 3a. The blue part is the cladding structure, the yellow part is the fiber core structure, the red part is the collapse region, and the green part is the air region. The light field distribution in the SHTSCF could be clearly observed. The structural parameters of the optical fiber are the same as those used in the experiment, as shown in Figure 2a. The refractive index of the fiber core, cladding and air hole are set to 1.450, 1.444 and 1, respectively. The injected light source is Gaussian. The incident light is set to propagate along the Z-axis, and the transmission wavelength was chosen as 1550 nm. The mode field distribution of the simulated light is shown in Figure 3b.

The simulation result clearly shows the distribution of the light field in the sensing structure. After the first collapse region, the light will diverge into the air core, the fiber core and the cladding, and the core mode in the fiber core and the cladding mode in the cladding will be excited. After the 3 mm-long SHTSCF, the light converges to interfere in the second collapse region. For the wavelength of 1550 nm, the energy is still mainly concentrated in the fiber core, cladding and air core, which means this wavelength is not the resonant wavelength. In particular, visible light energy can be observed in the fiber core. Consistent with the above analysis, part of the beam is transmitted in the cladding, core and pores to form the inline MZI, which satisfies the hypothesis of the sensing mechanism.

## 3. Spectral Analysis

The SHTSCF can be considered as a waveguide formed by a low-index core surrounded by a high-index cladding layer, and the anti-resonant effect in the layer can be regarded as a reflective Fabry–Perot (FP) like interferometer [24]. Therefore, a periodic and narrow loss drop corresponding to the resonant conditions of the FP cavity and high reflection occurs at the anti-resonant wavelengths that appear in the transmission spectrum. The wavelength of the lossy dip (resonant wavelength) can be given by equation [25]:

(1) $$\lambda_{k}=\frac{2h}{k}\sqrt{n_{2}^{2}-n_{1}^{2}}$$

where *h* is the cladding thickness of the SHTSCF, *n*_1_ is the refractive index of the air hole, *n*_2_ is the refractive index of cladding, and *k* is the order of the resonance. Besides, the intensity at the resonant wavelength is affected by the reflection coefficient on both sides of the FP, thus the transmission power corresponding to the resonate condition can be expressed as:

(2) $$T_{ARROW}=\frac{{\left(1-n_{1}n_{2}\right)}^{2}{\left(n_{1}+n_{2}\right)}^{2}}{1+n_{2}^{4}-2n_{2}^{2}}I_{ARROW}$$

where *T_ARROW_* is the transmission power at the resonant wavelengths, and *I_ARROW_* is the input light intensity at the resonant wavelength.

According to the previous analysis, in addition to the anti-resonant effect, the proposed structure also has an inline MZI. The normalized intensity transmission of the multimode interference can be expressed as:

(3) $$T_{MZI}=B_{m}\cos^{2}(\frac{\pi l}{\lambda }\cdot \Delta n_{m})$$

where *B_m_* represents the coefficient of the comb spectrum intensity, *m* is the order of the cladding mode, Δ*n_m_* is the effective refractive index difference between the core mode and cladding mode in *m* order, and *l* is the physical length of the SHTSCF cladding.

The interference components in the sensor can be analyzed according to the spectrum. The length of SHTSCF in the sensor will affect the contrast and free spectral range (FSR) of the MZI fringe. The FSR caused by the anti-resonance effect is usually tens of nanometers. When the SHTSCF is too long, the transmission distance of the excited cladding mode in the cladding is too long, which leads to the loss and smaller FSR and extinction ratio, affecting the measurement range and accuracy of the subsequent sensing experiments. When the SHTSCF length is too short, the large extinction ratio of MZI is beneficial to the sensing experiment, but the wide FSR will drown the resonance wavelength of the anti-resonance, making it impossible to distinguish. Based on the above considerations and experimental tests, the SHTSCF with a length of 3 mm was finally selected for subsequent sensing experiments. According to the FSR of the in-line MZI, the length of the SHTSCF can be chosen to be 3 mm, the FSR of which is around 1~2 nm.

**Figure 4:**
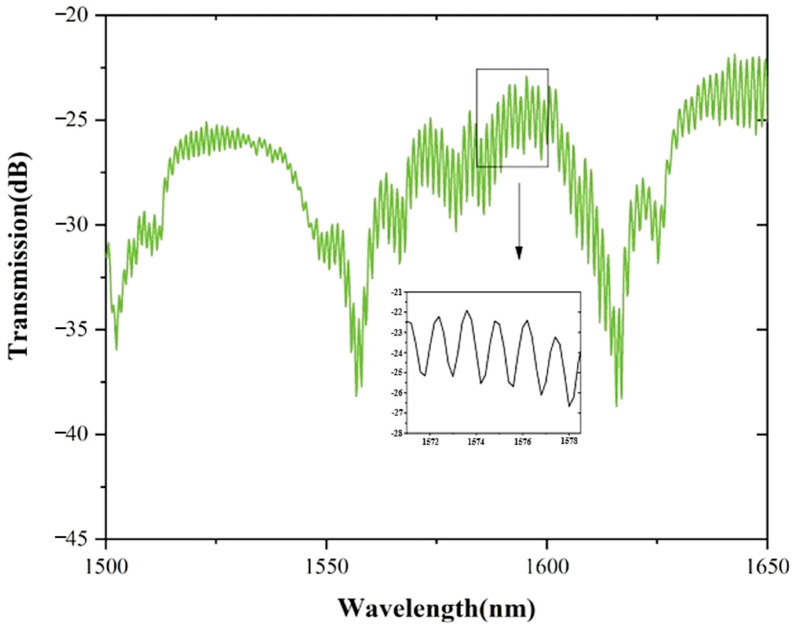
Experimental spectrum of the combinational two different mechanisms.

Splice both ends of SHTSCF with SMF directly to obtain the experimental spectrum, as shown in Figure 4. Obviously, the experimental spectrum has three dominant dips in the range of 1500 nm to 1650 nm produced by the anti-resonant effect and the comb spectrum is nonuniform owing to multimode interference generation in SHTSCF. The inset is a magnification of the spectrum in the resonance region from 1571 nm to 1578.5 nm.

**Figure 5:**
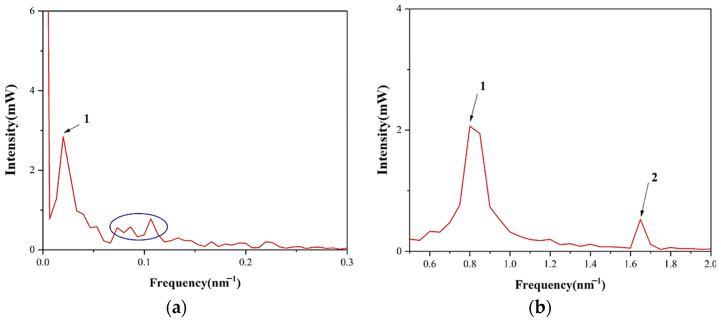
(**a**) Spatial frequency spectra from 0 to 0.3 nm^−1^; (**b**) Spatial frequency spectra from 0.5 nm^−1^ to 2 nm^−1^.

In order to analyze the composition of interference phenomena that occurred in SHTSCF, it is necessary to apply a fast Fourier transform (FFT) to the mixed spectrum. It is well-known that different spatial frequencies correspond to interference in different FSR. As shown in Figure 5a, the peak, marked as number 1, with a light intensity of 2.8 mW at the spatial frequency of 0.019 nm^−1^ corresponds to an FSR of 52.6 nm, which can be considered to represent the anti-resonant effect according to the estimated optical path difference. It also can be seen that there are several weak peaks around 0.1 nm^−1^ in the blue circle, which represents the irregular interference pattern in the transmission spectrum. In addition, two distinct peaks, marked as number 1 and number 2, exist at 0.8 nm^−1^ and 1.65 nm^−1^ when the frequency range is from 0.6 nm^−1^ to 2.0 nm^−1^, as shown in Figure 5b, which is caused by high-order cladding patterns involved in inline MZI. The peak at 0.8 nm^−1^ is dominant.

## 4. Experiments and Results Analysis

**Figure 6:**
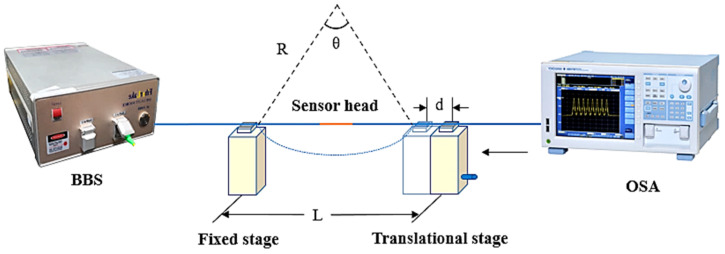
Diagram of experimental setup.

According to the analysis in the previous section, there are mainly two different mechanisms in the spectrum of the sensor head, which have the potential to achieve dual-parameter measurement. Therefore, a curvature and a temperature experiment are conducted to investigate the sensing properties. Figure 6 shows the experimental device for curvature sensing, fixing the 3 mm-long proposed sensor head between the two fiber clamps placed on two stages, respectively. One end of the sensor head is connected to a broadband light source (BBS), and the other end is connected to the Optical Spectrum Analyzers (OSA, Yokogawa AQ6370c, Musashino, Japan). One stage is fixed, and the other stage can be moved to bend the sensor region.

The curvature of the fiber can be changed by changing the distance between the two fiber clamps. The relationship between the radius of curvature and distance can be obtained from the following equation:

(4) $$R\sin(\frac{L}{2R})=\frac{L-d}{2}$$

where *L* is the distance between the two translation stages, *d* is the displacement of the SHTSCF, and *R* is the radius of curvature. In our experiment, *L* = 10 cm, step length is 10 μm to make *d* increase. Thus, the curvature is changed from 0.85 m^−1^ to 1.56 m^−1^. Because this equation is a transcendental equation, there is an implicit function that cannot be solved directly, so MATLAB is used to solve the change of curvature when the knob is turned once.

**Figure 7:**
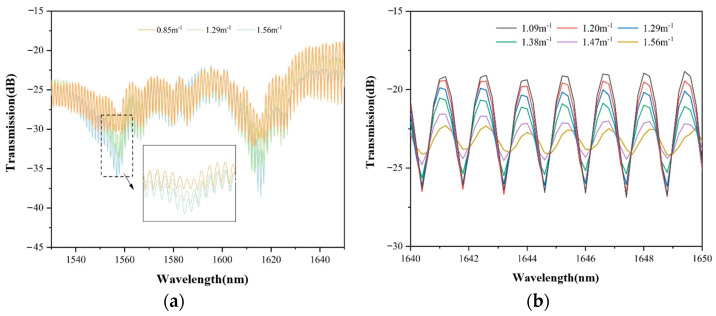
(**a**) Curvature response in the 1530~1650 nm spectral range; (**b**) Curvature response in the spectral range from 1640 to 1650 nm.

As shown in Figure 7a, in combination with the theory from the spectral drift in the range of 1530~1650 nm, with the increase in curvature, more light will escape from the cladding, and the intensity of wavelength at a certain dip in the experiment will decrease with the increase in curvature. The inset is a magnification of the intensity change in the curvature of 0.85 m^−1^, 1.29 m^−1^, and 1.56 m^−1^. The resonant wavelength caused by the anti-resonant effect is hard to distinguish because of the multimode interference, which means the multimode interference is loaded on the anti-resonant effect and makes the resonant wavelength have a bandwidth and ripple.

In order to measure the response of MZI and anti-resonance effect to curvature in the fiber, part of the spectrum is intercepted for analysis. Figure 7b shows the response of the MZI spectrum to curvature in the range of 1640~1650 nm. It can be seen that there are slight fluctuations in several dip wavelengths in the range. Only a displacement of the order of picometers per unit curvature is expected. From the dip changes within the selected range, the energy leakage degree of the fiber caused by bending is much greater than the change in the effective refractive index of the fiber, so the response of the spectrum to curvature is mainly reflected in the change in intensity. Due to the superposition of multiple mode interference effects in the fiber, the loss of the dip of MZI in different wavelength regions has different sensitivity responses to curvature but is generally wavelength insensitive.

**Figure 8:**
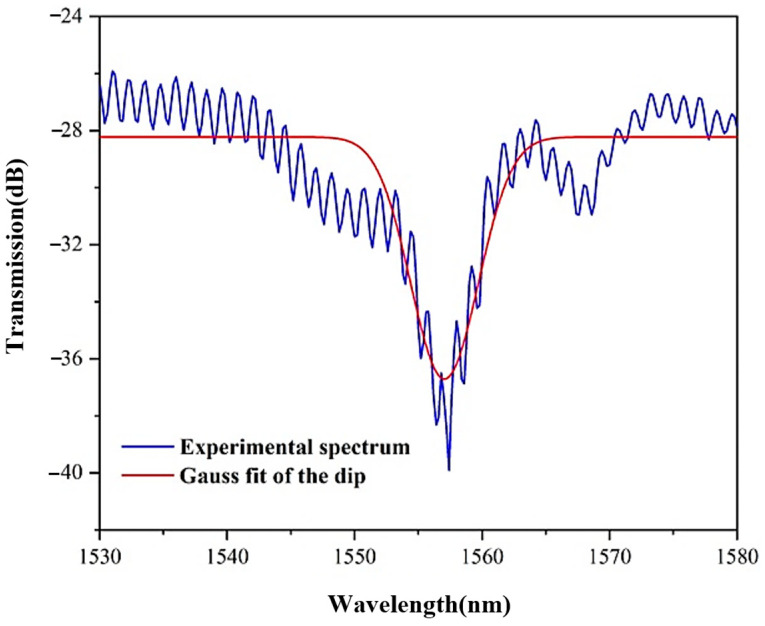
The Gaussian fitting of the dip wavelength.

To better lock the dip wavelength, we use a Gaussian fits method to obtain the resonant wavelength, as shown in Figure 8. Although there are errors in the fitting process, fitting errors of every resonant wavelength are the same, so the dip shift trend will not change in the sensitivity measurement. From the dip variation, the energy leakage due to bending is much greater than the change in the effective refractive index of the fiber. Therefore, the response of the interference spectrum to curvature is mainly manifested in the change of intensity. The intensity demodulation method can be used to measure the curvature, which can avoid the interference of temperature on the measurement results.

**Figure 9:**
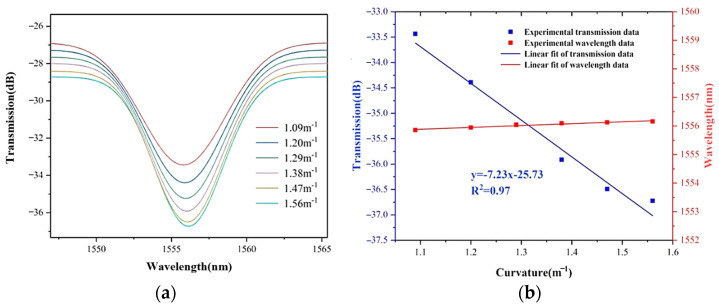
(**a**) The intensity Gaussian fit at the resonant wavelength from 1.09 m^−1^ to 1.56 m^−1^; (**b**) The linear fit of the resonant wavelength about the sensitivity of −7.23 dB/m^−1^.

Gaussian fitting is performed on the six sets of experimental data of different curvatures, 1556 nm is selected as the tracking wavelength, and the intensity drift trend of the resonant wavelength can be obtained. Figure 9a shows the Gaussian fitting curves of the spectrum at the same resonant wavelength with different curvatures.

The curvature sensitivity can be obtained by tracking the decrease point in Figure 9a, both the intensity and wavelength of the dip are fitted in Figure 9b. The sensitivity of −7.23 dB/m^−1^ can be obtained by linear fitting of the wavelength intensity when the curvature increases from 1.09 m^−1^ to 1.56 m^−1^. By the way, the wavelength shift of the dip is less than 0.2 nm, which can be regarded as a straight line, meaning that wavelength fluctuations can be ignored when the curvature is tested. In addition to the influence of environmental effects such as temperature, this fluctuation may also be related to the center wavelength drift of the light source.

**Figure 10:**
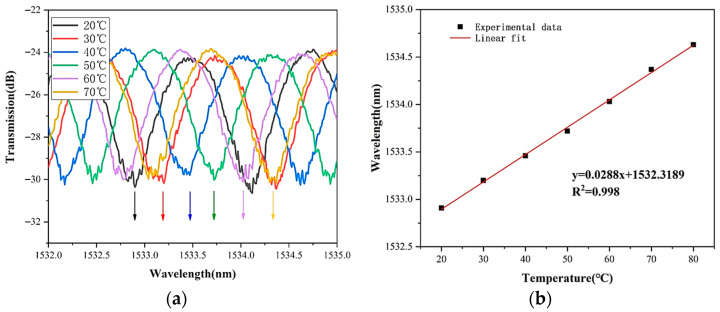
(**a**) The wavelength variation of the MZI dip with temperature increasing from 20 °C to 70 °C; (**b**) The linear fit of the MZI dip about the sensitivity of 28.8 pm/°C.

As for the temperature measurement, we put the sensor into a variable temperature groove by controlling the TEC module from 20 °C to 80 °C. In order to more intuitively measure the sensitivity of MZI and anti-resonance effect to temperature in the fiber line, the variation in part of the spectrum with the temperature at different temperatures is intercepted. Figure 10a shows the temperature drift of a certain MZI wavelength in the range of 1532~1535 nm. It can be seen from arrows of different colors in the figure. The interference dip shifts to the long wavelength direction with increasing temperature. Figure 10b shows the linear fitting of eight groups of data points, and the results show that the temperature sensitivity is 28.8 pm/°C.

**Figure 11:**
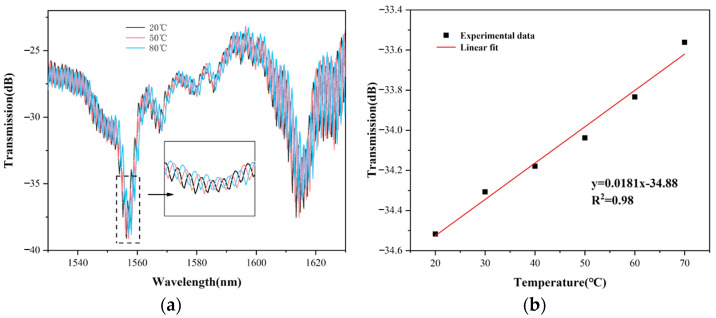
(**a**) The variation of the transmission spectrum with temperature increasing from 20 °C to 80 °C; (**b**) The linear fit of the resonant wavelength about the sensitivity of 0.018 dB/°C.

By observing the variation in the dip wavelength of the anti-resonance effect, it can be found that the intensity of the anti-resonance wavelength is almost not affected by temperature, as shown in Figure 11a. The inset is an enlarged view of the dip shift with temperature change. Gaussian fit is also used in the temperature measurement to extract the resonant wavelength, and the linear fit of the experimental data in Figure 11b indicates that the temperature sensitivity of the resonant wavelength intensity is −0.018 dB/°C. This shows that when using intensity demodulation of a resonant wavelength for curvature sensing, the temperature cross-sensitivity as low as 0.0025 m^−1^/°C can be achieved.

The intensity of a resonant wavelength in the anti-resonant effect is caused mainly by the curvature change; the wavelength shift of the MZI dip is caused by the temperature change. Thus, the curvature change of the sensor can be obtained by the intensity of the resonant wavelength in the transmission spectrum, and the temperature change can be obtained by the wavelength shift change of the MZI. There is almost no interference between wavelength demodulation and intensity demodulation, so dual-parameter measurement of temperature and curvature can be achieved by using these two mechanisms simultaneously.

## 5. Conclusions

In summary, a novel optical fiber intrinsic sensor based on the anti-resonance effect combined with inline MZI is proposed and experimentally verified. The SHTSCF is part of the designed sensor, which is only 3 mm in length. The sensor can monitor curvature and temperature simultaneously, and the sensitivity is with very little crosstalk. The curvature sensitivity of −7.23 dB/m^−1^ can be obtained by Gaussian fitting the intensity variation at the dip wavelength. Meanwhile, by tracking the shift of the MZI dip wavelength, the temperature sensitivity is 28.8 pm/°C. In addition, the cross-sensitivity of temperature to curvature for Gaussian fits dip is about 0.0025 m^−1^/°C, which represents a dual-parameter measurement with very little interference. Overall, the proposed sensor head has advantages including a miniature structure, ease of fabrication, and high integration. It is believed to have a bright application prospect in structural health monitoring in many narrow application scenarios.

## Data Availability

Data are contained within the article. More specific data are available from the corresponding author upon reasonable request.
